# Refining methods for attributing health impacts to climate change: a heat-mortality case study in Zürich

**DOI:** 10.1007/s10584-025-04011-5

**Published:** 2025-09-10

**Authors:** Rupert F. Stuart-Smith, Ana M. Vicedo-Cabrera, Sihan Li, Friederike E.L. Otto, Kristine Belesova, Andy Haines, Luke J. Harrington, Jeremy J. Hess, Rashmi Venkatraman, Thom Wetzer, Alistair Woodward, Kristie L. Ebi

**Affiliations:** 1 Oxford Sustainable Law Programme, Smith School of Enterprise and the Environment, University of Oxford, Oxford, OX1 3QY UK; 2 Institute of Social and Preventive Medicine, University of Bern, Bern, Switzerland; 3 Oeschger Center for Climate Change Research, University of Bern, Bern, Switzerland; 4 School of Geography and Planning, University of Sheffield, Sheffield, S3 7ND UK; 5 The Grantham Institute for Climate Change, Imperial College London, London, SW7 2AZ UK; 6 Department of Primary Care & Public Health, School of Public Health, Imperial College London, W6 8RP London, UK; 7 Centre on Climate Change and Planetary Health, London School of Hygiene & Tropical Medicine, London, WC1H 9SH UK; 8 Department of Public Health, Environments and Society, London School of Hygiene & Tropical Medicine, London, WC1H 9SH UK; 9 Te Aka Mātuatua School of Science, University of Waikato, Hillcrest, Hamilton, 3216 New Zealand; 10 Department of Environmental and Occupational Health Sciences, University of Washington, Seattle, WA 98195 USA; 11 Faculty of Law, University of Oxford, Oxford, OX1 3UL UK; 12 Section of Epidemiology and Biostatistics, School of Population Health, University of Auckland, Auckland, 1010 New Zealand; 13 Department of Global Health, University of Washington, Seattle, WA 98195 USA; 14 School of Geography and the Environment, University of Oxford, South Parks Road, Oxford, OX1 3QY UK

**Keywords:** Climate change attribution, Epidemiology, Climate impacts, Health.

## Abstract

Heat-related deaths occur throughout the summer months, peak during heatwaves, and are affected by temperature and exposed populations’ sensitivities to meteorological conditions. Previous studies found that climate change is increasing heat-related mortality worldwide. We build on existing epidemiological methods to shed light on the adverse effects of climate change on human health. We address limitations in existing methods and apply refined approaches to assess heat mortality attributable to human-induced climate change in Zürich, Switzerland, over 50 years (1969–2018) including a case study of summer 2018. Our methodological refinements affect how counterfactual climate scenarios are derived, and facilitate accounting for changing vulnerability, and assessing impacts during and outside heatwaves. We find nearly 1,700 heat-related deaths attributable to human-induced climate change between 1969 and 2018. Declining vulnerability to heat avoided at least 700 heat-related deaths. The approach described here could be applied elsewhere to quantify the effect of climate change on other health outcomes.

**Supplementary Information:**

The online version contains supplementary material available at 10.1007/s10584-025-04011-5.

## Introduction

Human-induced climate change causes substantial morbidity and mortality from increasingly frequent and intense extreme weather events, rising temperatures and sea levels, and changes in rainfall, among other factors (Cissé et al. 2022; Romanello et al. 2024). These physical (weather and climate-related) hazards have direct physiological effects, shift the distribution of pathogens and disease vectors, reduce the yields and nutrient quality of food crops, and influence socioeconomic determinants of health. As global temperatures rise, the magnitude of associated health impacts is projected to increase further (Ebi et al. 2018; Haines and Ebi 2019; Watts et al. 2021). Studies quantifying the already-occurring effects of climate change on health can improve awareness of its impacts, inform adaptation decisions and political negotiations around loss and damage (James et al. 2019), and provide evidence for climate-related lawsuits (Stuart-Smith et al. 2024). Despite their relevance, attribution studies of this kind remain limited in number, scope, regional coverage, and in the rigour of methods applied: a recent review identified twenty studies that estimated the present-day health impacts of climate change (Carlson et al. 2025).

Current methods in attribution of the health impacts of anthropogenic climate change rely on established and widely applied approaches. Recent studies extended attribution analyses from assessing climate change influence on meteorological events to their health impacts, including on heat-related mortality (Mitchell et al. 2016; Mitchell 2016; Clarke et al. 2021; Vicedo-Cabrera et al. 2021) and the spatial extent of vector-borne disease (Gibb et al. 2023). These contributions combined the latest developments in climate trends and extreme weather event attribution with advanced methods in climate epidemiology to derive robust estimates of climate-related health impacts attributed to human activities (Vicedo-Cabrera et al. 2019, 2021). However, several methodological questions arise when integrating climate science and health methods that have not been sufficiently addressed or discussed. These include (1) how the counterfactual climate scenarios are derived and combined with epidemiological data and methods, (2) the assumptions behind the use of different definitions of vulnerability, and (3) the comparison between impact estimates during and outside extreme event periods (e.g., heatwaves).

Here, we identify and explore approaches for resolving these key methodological issues in health impact attribution studies and their application in attribution studies for specific events or trend studies. As an illustrative example of the application of these methods, we present a case study of the effect of climate change on heat-related mortality in the canton of Zürich (Switzerland, population 1.5 million in 2018) (Federal Statistical Office 2024). We use the case study to discuss the advantages, disadvantages and suitability of different approaches for calculating counterfactual temperatures. We also show the impact of accounting for changes in vulnerability in the impact model and discuss the assumptions behind each choice (Sect. 3.2). We define ‘vulnerability’ based on the exposure-response association, and therefore in the IPCC’s definition, our usage encompasses both the propensity or predisposition to be adversely affected (‘vulnerability’) and ‘exposure’, the presence of people in settings that could be adversely affected (Möller et al. 2023). Finally, we narrow down the investigation to the summer of 2018 to compare the attributable heat-related mortality accrued during a heatwave period with lengthier periods of milder summer temperatures (Sect. 3.3).

## Methods

**Fig. 1:**
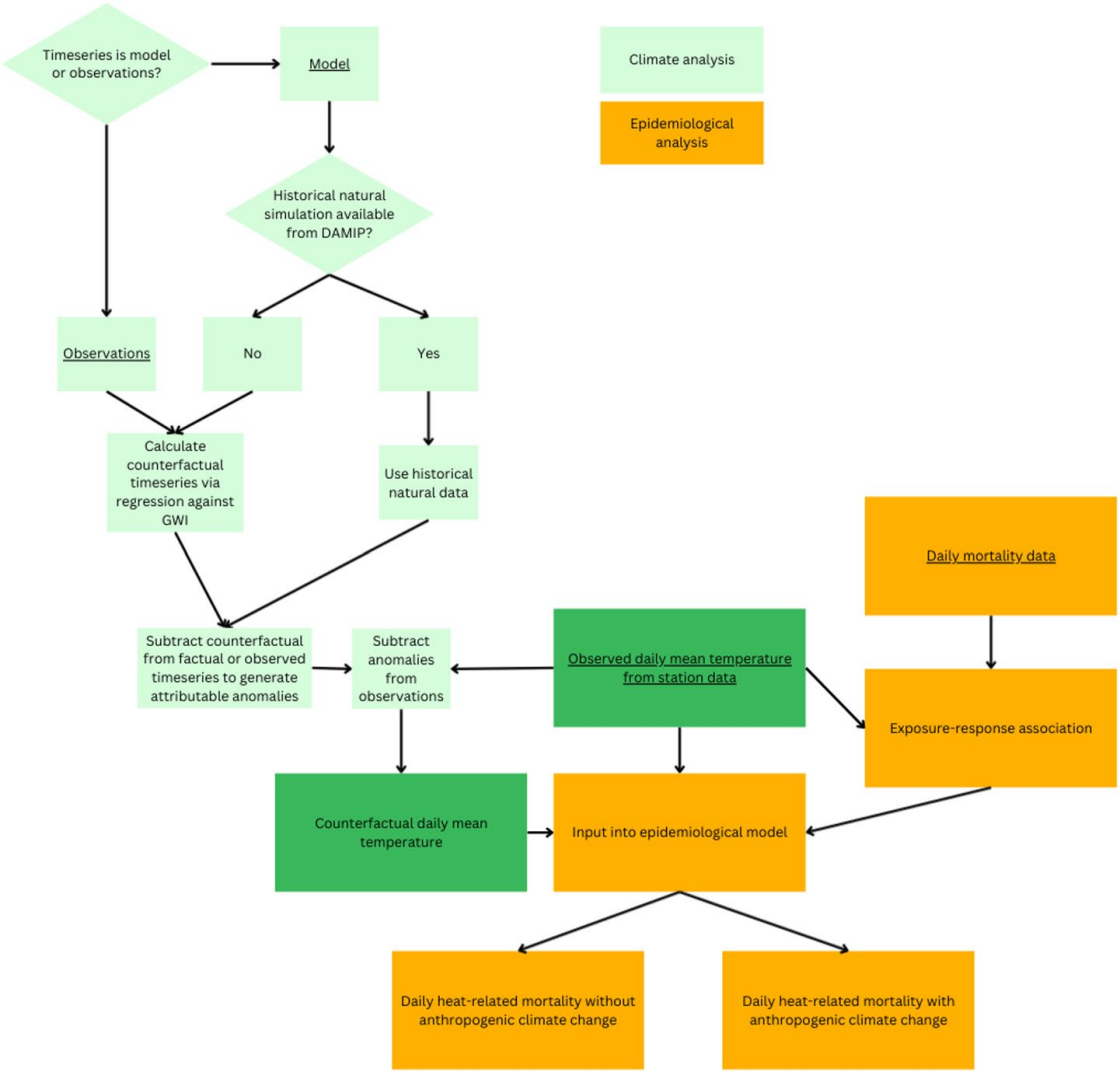
Process chart showing the main steps of the climate and epidemiological analyses carried out. Input data are underlined. Data input into the epidemiological model are shown in dark green

We collected observed daily mean temperature and all-cause mortality data in the Canton of Zürich between 1969 and 2018 (see data availability). We then quantified the daily number of heat-related deaths using the total mortality for each day under observed and counterfactual conditions. Fig. 1 provides an overview of the methodological framework, which is broadly in line with recently published guidance for health attribution research (Ebi et al. 2025). We calculated climate-change-attributable heat-related mortality as the difference between the mortality associated with observed and counterfactual temperatures.

### Constructing the counterfactual temperatures

To conduct a climate change-health attribution analysis, studies need meteorological inputs to the health model under two contrasting conditions, often a ‘factual’ scenario representing the observed climate, and ‘counterfactual’ conditions that exclude drivers of interest, such as the effect of anthropogenic greenhouse gas and aerosol emissions. Previous research used bias-corrected climate model data to represent both the historical climate, with anthropogenic and natural forcings (e.g., volcanic), and the counterfactual, ‘historical-natural’ climate from which anthropogenic climate forcings are excluded (Vicedo-Cabrera et al. 2021). However, while the summary distributions of observed and climate model data for extended periods are comparable, daily climate model data do not align with observed conditions; for instance, days with highest temperatures differ between models and observations (Fig. S1). For long-term studies where it is not necessary to precisely reproduce the conditions on specific days, this is of little consequence, and so in previous studies of this type (Vicedo-Cabrera et al. 2021) it was appropriate to use mean mortality for the day of the year, as is common in health impact projection studies (Vicedo-Cabrera et al. 2019), rather than observed data as the denominator to derive the daily fraction of deaths attributable to heat (Vicedo-Cabrera et al. 2021). However, in health attribution studies of single events (such as the 2003 heatwave in Paris) it would not be appropriate to use average mortality or raw/bias-corrected climate model output where observed conditions need to be accurately reproduced.

A modelling approach that accurately assesses the impacts of climate change on daily heat-related mortality is therefore needed. To do so, we derive counterfactual temperatures by subtracting the human-induced temperature change (due to anthropogenic greenhouse gas and aerosol emissions) for each day from the observations, based on climate-model and observation-based (in-situ and reanalysis) datasets. The counterfactual series therefore retains the pattern and variability of temperature found in the observations. This approach is similar to that previously applied (with annual data) to quantify economic impacts of climate change (Diffenbaugh and Burke 2019) but we are unaware of prior application of this approach to health impacts. The detrending method described in Mengel et al. (2021) removes the long-term trend in observed data to generate a counterfactual timeseries and similarly retains the observed pattern of meteorological values in the factual and counterfactual series (i.e., heatwaves occur at the same time in the two series) but represents the impact of observed climate change (Mengel et al. 2021). Through the use of the Global Warming Index, our method removes the change in temperature attributable to anthropogenic greenhouse gas and aerosol emissions, rather the trend in observed temperatures only. Moreover, our approach combines lines of evidence from observations and climate models, rather than trends in reanalysis only. Adapting the widely applied probabilistic attribution approaches described in Philip et al. (2020) and van Oldenborgh et al. (2021) the attributable temperature change is the difference between factual and counterfactual temperatures with probabilities equal to the observations, from daily climate model and reanalysis (observation-based gridded climate data) data. In our example, Summer (June-August) warming attributable to anthropogenic influence in Zürich increased across the study period, reaching 1–1.5 °C in 2009–2018 in model simulations, and slightly higher in reanalysis datasets (Fig. S2).

The main advantage of counterfactuals calculated by subtracting the attributable change from an observational timeseries, relative to simulations of counterfactual temperature series taken directly from climate models (e.g., simulations from the Detection and Attribution Model Intercomparison Project, DAMIP (Gillett et al. 2025), is that it replicates observed events on the dates at which they occurred and thus can be used to analyse both long-term impacts and those of individual weather events. Additionally, this allows us to use observed daily mortality to derive the daily heat-related deaths rather than mean mortality for the day of the year, avoiding spikes in mortality typically found in observed series being spuriously attributed to temperature on days when model temperatures were not high (Vicedo-Cabrera et al. 2021). Mean values by day of the year retain seasonal patterns and capture long-term trends but do not represent the full day-to-day variability of mortality. Thus, the use of observation-based counterfactuals, combined with observed mortality, affords greater confidence in our estimation of absolute values of heat-related mortality (Table S3), especially in short periods of time (i.e., a specific summer season) and allows us to account for population changes over time. The effect of this approach can be seen in Fig. S1, where in our analysis daily temperatures in both factual and counterfactual timeseries correspond to peaks and troughs in mortality, whereas GCM output (dashed lines in Fig. S1) does not rise and fall in step with observed mortality fluctuations.

Counterfactual temperatures were calculated by subtracting the temperature anomaly attributable to anthropogenic greenhouse gas and aerosol emissions from observed (station) temperatures for Zürich (Fluntern; see data availability statement). Here we describe how the attributable anomalies were calculated based on observations, reanalysis and climate model data. We calculated the change in daily-mean temperature attributable to anthropogenic climate change as the difference between the modelled daily-mean temperatures in the historical (1969–2018) and counterfactual climates in climate-model and observation-based (reanalysis) simulations.

#### Quantifying probability of observed temperatures

For each day of the study period, we calculated the probability of the observed temperatures. We do so by first detrending the observed temperatures by regressing against 4-yr smoothed global-mean surface temperature (GMST) using the NASA-GISS temperature dataset (Lenssen et al. 2019) and subtracting the product of the regression coefficient ($\:\alpha\:$) and GMST from the observations $\:\left(\alpha\:=\left(d{T}_{obs}/dt\right)/\left(d{T}_{GMST}/dt\right)\right)$. We then fitted the detrended temperatures to Logistic distributions that represented the distribution of temperatures in the 30-year period centred on the year in question, or for data near the end of the timeseries, the longest period that could be centred on the year in question. To do this, we set the mean of the distribution to the thirty-year (or longest possible) moving mean centred on the year in question. This ensures that the estimated probabilities of daily temperatures are consistent with the climate in which they occurred. We note that years near the end of the timeseries will be more affected by internal variability due to the shorter period used to estimate the mean of the temperature distribution.

The logistic distribution was chosen by fitting the detrended observed daily mean temperatures (with seasonal cycle removed by subtracting the 30-day moving mean) to commonly used statistical distributions and applying the Anderson-Darling and Kolmogorov-Smirnov tests to identify the distribution that gave the best fit to the data. The statistical distributions attempted were the Generalised Extreme Value (GEV), Logistic, Normal, Rayleigh, and t Location Scale distributions. The seasonal cycle was reinstated through the use of observations in constructing the counterfactual temperatures.

#### Analysis of gridded data

Model and reanalysis temperatures are taken from the grid cell containing Zürich (based on a nearest-neighbour selection); for the model temperatures, these are taken from an 18-member ensemble of Coupled Model Intercomparison Project Phase 6 (CMIP6) models detailed in Table S1. These models are all those with daily data available in The Centre for Environmental Data Analysis (CEDA) data archive at the time that the analysis commenced. We evaluated the models to assess whether the statistical characteristics of temperatures in the models were consistent with those in the observations by comparing the scale and shape parameters of each of the model fits with that of the observations. Following Philip et al. (2020), the models for which the 5–95% confidence intervals of these parameters of the statistical fits overlapped with the 5–95% confidence intervals of the parameters of the observations, were initially selected for use in the analysis. Because only one model passed this evaluation step, and it is preferable to include multiple models to better account for systematic uncertainty in the model representation of the climate system, due to model structural biases and differing representations of physical processes, we expanded the range of parameter values used to evaluate models slightly (by 5% relative to the 5 and 95% confidence intervals). This increased the number of models passing the evaluation step to seven.

Since our analysis relies primarily on the long-term temperature trend in the models rather than day-to-day temperature variability (which is taken from station observations) to estimate counterfactual temperature values, a slight broadening of the criteria for models to pass the evaluation step has limited consequence for the results. For the five models included for which historical-natural simulations were unavailable, only the long-term trend is used to estimate counterfactual values, which therefore display no sensitivity to the shape of the temperature distribution. This justifies the inclusion of all seven models in our analysis, and this is further supported by the fact that all seven selected models produce warming attributable to human influence that, at the end of the series, lie within the range of the observation-based datasets (Fig. S1). Our model evaluation means that the sample we use successfully simulate the temperature variability of the Swiss climate.

After selecting the models for use in the analysis, the equilibrium climate sensitivities of selected models (Tables S1, S2) are compared with the full set of CMIP6 models. The mean climate sensitivity of the selected models is very close to that across CMIP6 models, and the selected models also capture a substantial portion of the range in climate sensitivity found across CMIP6 models (Table S8) indicating that the sample we are left with is representative of the full set of CMIP6. For models for which we Had historical-natural simulations, the temperatures under historical and historical-natural conditions, both for 1969–2018, were estimated as the temperatures with the same probability as the temperature of the same day in the observations. For other models, and the observations and reanalysis datasets, we calculated annual temperature anomalies attributable to anthropogenic climate forcing using a separate method, detailed below.

Following the selection of models as detailed above, we then proceed with the calculation of counterfactual temperatures. First, the difference in temperature caused by the difference between the mean elevation of the model grid cell (the spatial resolutions of which range from 0.94° x 0.94° to 1.875° x 1.25° and are stated in Table S2) and the station observations is corrected for using a lapse rate of −6.5 °C km^−1^, consistent with summer lapse rates found elsewhere in the European Alps (Zemp et al. 2007; Nigrelli et al. 2018) (while the precise lapse rate will vary with humidity, this does not affect our overall results since only attributable anomalies rather than absolute temperature values are used in our model analysis). The same approach was used for the reanalysis data, for which we use three climate reanalysis datasets: the Modern-Era Retrospective analysis for Research and Applications, Version 2 (MERRA-2) (Global Modeling and Assimilation Office (GMAO) 2015), Berkeley Earth Surface Temperature Project (Rohde and Hausfather 2020), and ERA5 (Hersbach et al. 2020; Bell et al. 2021). We then detrended the model and observation-based temperatures by regressing against each model and reanalysis timeseries’ GMST and removed the seasonal cycle in the same manner as the station observations. We then fit the resulting timeseries to the same statistical distribution as the observations. The distribution was shifted over time such that it reflected the temperatures of the 30-year period centred on the year in question using the same approach as the station observations.

#### Calculating counterfactual temperatures

We then calculated the attributable change in temperature for each day in all models that pass the evaluation step. We used the selected ensemble of CMIP6 models to simulate the distribution of daily temperatures in the historical period and in the absence of anthropogenic greenhouse-gas and aerosol emissions. We used daily output from historical (1850–2014) and SSP 5-8.5 (2015 onwards) runs as transient simulations (because the projections are only used to generate distributions of temperature until 2018, results have limited dependence on scenario choice as all SSPs are very similar until ~ 2030). For models for which both transient and historical-natural simulations (from the Detection and Attribution Model Intercomparison Project, DAMIP (Gillett et al. 2016) were available, the attributable change in temperature was calculated as the difference between the temperatures in the two sets of simulations, for probabilities equal to those in the observations. The probabilities of historical and historical-natural model temperatures were calculated using the same approach taken for the observations, with probabilities calculated based on a distribution representing the 30-year period centred on the calendar year in question.

For the models for which historical-natural simulations were unavailable, the transient simulations were used and the distribution of daily-mean temperatures in the absence of human influence estimated by shifting the mean of the temperature distribution by the product of the change in GMST attributable to anthropogenic climate influence and a scaling factor. Global-mean human-induced warming is taken from the Global Warming Index, an estimate of the anthropogenic contribution to global externally forced temperature change (Haustein et al. 2017). The scaling factor is dataset and region-specific and is the regression co-efficient calculated in detrending the model/observational temperature data, which is the ratio between the gradients of local temperature observations and (4-yr smoothed) GMST from NASA-GISS (Lenssen et al. 2019). These two methods for calculating counterfactual temperatures are both commonly used in attribution studies when historical-natural simulations are available for some models but not others (Philip et al. 2020). Multiple reanalysis datasets were used to account for variations between the trend in temperature (and therefore estimated attributable warming) across observational products (Perkins-Kirkpatrick et al. 2024).

The regression-based method used for timeseries for which historical-natural simulations were unavailable can effectively account for the effect of anthropogenic influence on the climate on multi-decadal to centennial scales. However, it does not account for interdecadal variability. Consequently, this approach does not capture short-term influences on region-specific warming, such as Lowered central European temperatures in the 1970s due to anthropogenic aerosol emissions (Wilcox et al. 2013; Undorf et al. 2018) and so may overestimate temperature anomalies attributable to anthropogenic influence in this period, as seen in Fig. S2.

This approach is also based on three assumptions. First, using GMST as a covariate to represent anthropogenic influence on Local temperatures assumes that the Long-term proportional contributions of anthropogenic and natural forcing to temperature change are equal at local and global scales, and therefore that there are no independent local factors that could create century-scale local climate trends. Such local factors might include local changes in albedo due to land-cover alterations over the past 150 years. Analyses of the effects of land use and land cover change found limited changes in mean temperature and slight cooling in summer months over central Europe in the historical period, masking some of the attributable warming trend and rendering our results conservative (Zhang et al. 2024). No other factors that could have caused century-scale climate trends are known for this region, such as volcanic activity with effects localised to Switzerland. A similar consideration of the possible role of local factors that could cause century-scale climate trends should be given if applying these methods to other locations. A similar assumption applies to the use of gridded climate data to calculate attributable changes in temperatures: local factors are assumed not to cause local century-scale climate trends to differ significantly from trends at the scale of the grid cells. This is a widely applied assumption in climate change attribution studies (Philip et al. 2020) that we adopt here. Moreover, since our analysis is at the Canton scale (rather than a finer, such as city, scale) our results are less sensitive to this concern since this larger area is, on average, better represented by a grid cell than would be a specific location. However, location-by-location differences within the grid cell may be present.

Secondly, a common assumption in climate change attribution studies is that the trend in temperature extremes shifts with GMST, and that the scale parameter of the temperature distribution is unchanged over time. This is well supported for studies using large ensembles of model simulations (Philip et al. 2020). Nevertheless, we tested this assumption by evaluating the sensitivity of attributable mortality to the values of the scale parameter. We iteratively fit 30-yr windows of detrended data to the statistical distribution and found that mortality is unchanged (< 0.01% change) for a 1 standard deviation shift in the values of the parameters.

Thirdly, our analysis is predicated on an assumption that the attributable shift in temperature is constant between the summer and the full year. This is approximately true in Europe, where summer temperatures rose at 0.0251 °C/yr between 1951 and 2020, compared with an annual warming rate of 0.0267 °C/yr (0.0556 °C/yr in summer vs. 0.0551 °C/yr annual warming over 1985–2020) (Twardosz et al. 2021).

#### Case study: summer of 2018

To date, most climate change attribution studies that focus on meteorological conditions evaluate the effects of climate change on individual extreme weather events, such as heatwaves. However, in epidemiology, longer-term studies at the seasonal (Ballester et al. 2023) or multi-annual (Vicedo-Cabrera et al. 2021) timescale are more common. While peaks in heat-attributable mortality occur during heatwave periods, focusing only on extreme temperatures may not capture the full extent of the impacts of climate change. We therefore assess heat-related mortality within and outside of heatwaves by limiting our analysis to the summer of 2018 and quantified heat-related mortality based on observed and counterfactual temperatures.

### Epidemiological analysis

Our epidemiological analysis largely replicates the approach applied by Vicedo-Cabrera et al. (2021), whose method was developed in Gasparrini and Leone (2014), with several extensions. We calculated counterfactual heat-related mortality using the counterfactual temperatures generated as described in Sect. 2.1 above.

**Fig. 2:**
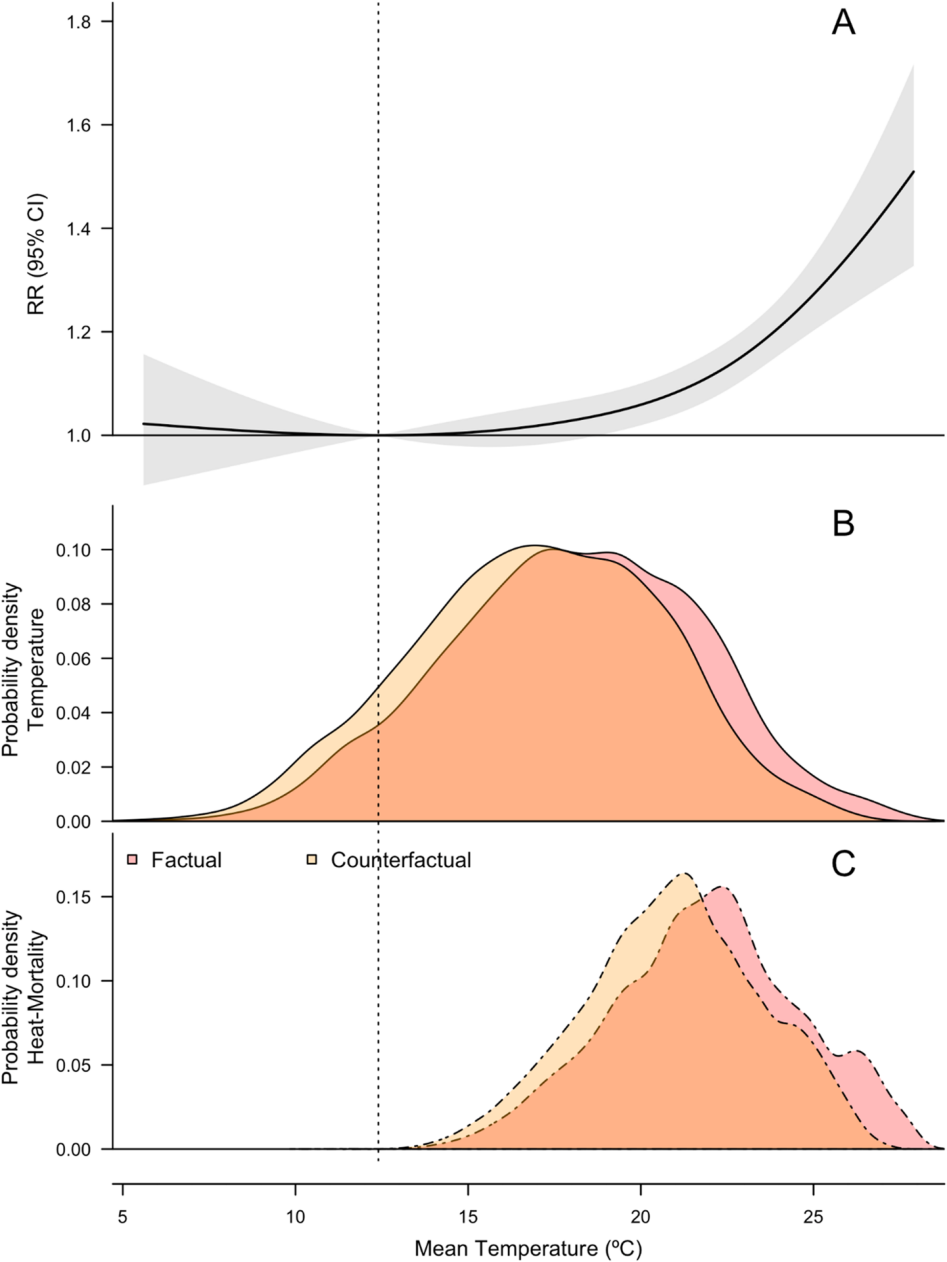
Exposure-response association for the Canton of Zürich, and probability density functions for temperatures under factual and counterfactual conditions, and the resulting distribution of mortality rates. (**A**) exposure-response association calculated using observed temperature and mortality data for 1969–2018, and the 5–95% empirical confidence intervals (shaded area). (**B**) PDFs of observed temperatures and a synthesis of model and observation-based counterfactual temperature timeseries (Methods). (**C**) Proportion of heat-related mortality occurring at different temperatures. The vertical dashed line shows the minimum mortality temperature.

Here we provide a brief overview of the epidemiological methods applied, noting elements of the methods specific to our study. We estimated the temperature-mortality relationship (Fig. 2) by conducting a time-series analysis with generalised linear models and quasi-Poisson regression using observed daily mean temperature and mortality data (see ‘Data availability’) during June-August (Vicedo-Cabrera et al. 2021) although we note that heat events and some limited heat-related mortality may occur outside of these months and are not accounted for. This renders our results conservative. The analysis was performed across the whole study period (June-August 1969–2018) to obtain the overall exposure-response relationship and the same approach repeated for three subperiods (1969–1985, 1986–2003, and 2004–2018) to assess the impacts of changing vulnerability to heat. Daily mean temperature is widely used as a determinant of heat exposure in calculating temperature-mortality association, and by subperiod to obtain the corresponding association estimates relationships (Gasparrini et al. 2015a; Vicedo-Cabrera et al. 2019, 2021; Lee et al. 2020).

We modelled the temperature-mortality dependency with a distributed lag non-linear model that simultaneously accounts for delayed effects and non-linearity of the association, typically found in temperature-health studies. Specifically, the exposure-response dimension was defined with a natural spline with two internal knots placed in the 50th and 90th percentile of the summer temperature distribution. The lag-response dimension also included a natural spline with two internal knots equally spaced in the Log scale and up to 7 days of lag. The time-series model included a natural spline of day of the year with 4 degrees of freedom and an interaction by year, and a natural spline of time with 1 knot every 10 years which affords sufficient statistical power to control for both seasonality and long-term trends. The specifications of the time series model and the definition of the cross-basis function of temperature are the ones described in Vicedo-Cabrera et al. (2021) and were used extensively in previous assessments (Gasparrini et al. 2015a, b; Sera et al. 2020; Vicedo-Cabrera et al. 2021). We did not control for air pollution and/or humidity because previous assessments suggested that the role of these variables as confounders was negligible (Gasparrini et al. 2015a; Armstrong et al. 2019).

Observation-based estimates of the exposure-response association between temperature and mortality using a time-series regression and distributed lag non-linear model is the state-of-the-art epidemiological method for assessing health impacts of environmental stressors (Gasparrini et al. 2017; Vicedo-Cabrera et al. 2021; Lo et al. 2022). We used this association to calculate the heat-related mortality fraction for each day when temperatures exceeded the temperature of minimum mortality under both observed conditions and counterfactual temperatures with the effect of anthropogenic greenhouse gas and aerosol emissions excluded (see Methods, ‘Constructing the counterfactual temperatures’). The minimum mortality temperature is the temperature at which risk of death is Lowest across the full observational timeseries. We quantified the uncertainty of the estimates by generating 1,000 samples of the coefficients defining the exposure-response association through Monte Carlo simulations, assuming a multivariate normal distribution. We derived the 95% empirical confidence intervals (eCI) from the resulting distribution, corresponding to the 2.5th and 97.5th percentiles. The eCI of the ensemble estimates of the counterfactual scenarios (i.e., by averaging across the single-series estimates) were estimated by combining the single-series distributions. Thus, in this way we account for both uncertainty of the exposure-response function and the variability across the different counterfactual series.

## Results

### Constructing the counterfactual temperatures

**Fig. 3:**
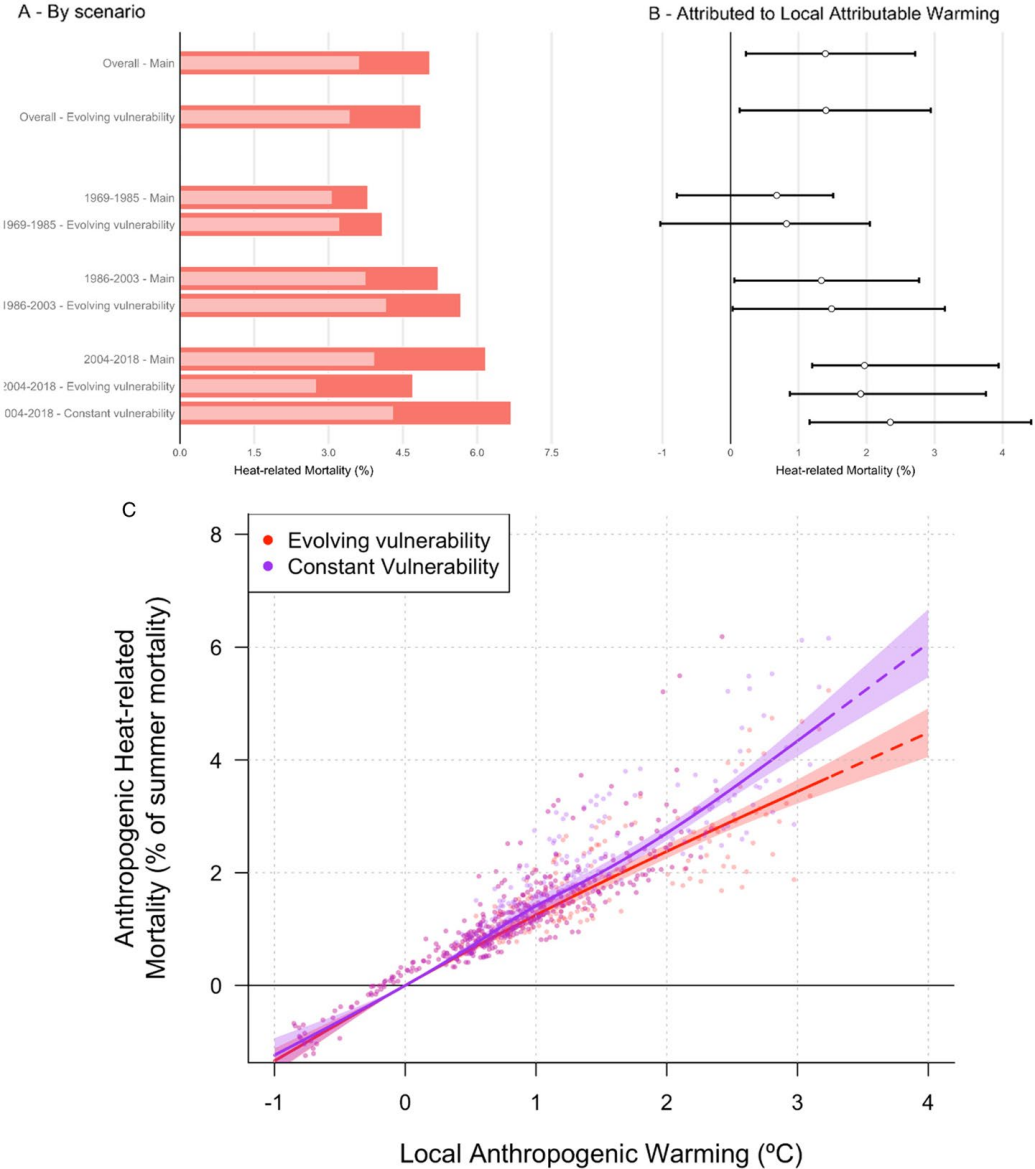
Heat-related mortality attributable to regional anthropogenic climate change for the Canton of Zürich. (**A**) heat-related mortality as a % of all-cause summer mortality for 1969–2018 (overall exposure-response and ‘evolving vulnerability’ scenario only) and for each of the three time periods considered in the analysis. The ‘evolving vulnerability’ and ‘constant vulnerability’ scenarios were identical for 1969–1985 and 1986–2003 so only ‘evolving vulnerability’ is shown. Dark red bars show the heat-related mortality under observed temperature conditions, lighter bars are the mean of the counterfactual scenarios. (**B**) as in panel **A** but showing the heat-related mortality attributable to climate change, with 5–95% empirical confidence intervals. (**C**) the relationship between the regional temperature anomaly attributable to anthropogenic climate change (for Zürich) and the attributable heat-related mortality as a percentage of all-cause summer mortality for the ‘evolving vulnerability’ (red) and ‘constant vulnerability’ scenarios (violet). The trend lines are calculated as a natural spline with 3 degrees of freedom, which produced the best fit to the data of the fits attempted (linear, and natural spline with 3, 4 and 5 degrees of freedom). Dashed lines show the continuation of the fit at temperature anomalies for which no data are available. We note that the ‘constant vulnerability’ scenario only excludes the change in exposure-response association between 1986–2003 and 2004–2018, and that further adaptation would be expected to occur in response to further warming, reducing the sensitivity of mortality risk to temperature. The shaded area represents the 5–95% confidence intervals of the trend. All values given are the synthesis values of all models and observation-based datasets.

Our analysis finds that between 1969 and 2018, 6,091 (2,716-9,476, 95% empirical confidence interval (eCI)) heat-related deaths occurred in the Canton of Zürich. In the counterfactual scenarios, heat-related mortality is 4,360 (988-7,931, 95% eCI; values are a synthesis of model and observed datasets, see Methods) leaving 1,683 (270-3,279) deaths attributable to anthropogenic climate change (Table S3). This is equal to 27.6% of heat-related mortality and 1.4% (0.2–2.7%) of summer all-cause mortality in Zürich over this period (Fig. 3b). We compared the results using counterfactual series derived from the seven climate models that passed our evaluation step, station observations, and reanalysis. The central estimate of attributable mortality across models ranges from 314 to 1,871, with higher attributable mortality estimates found in reanalysis and observation datasets, associated with stronger warming trends than indicated by the models (Table S3 and S4). This suggests the high sensitivity of the attributed burden to the amount of attributable warming and underlines the importance of using multiple datasets for estimating attributable changes.

### Accounting for changes in vulnerability

Previous epidemiological analyses of heat-related mortality attributable to climate change typically used the same vulnerability estimate (i.e., exposure-response association) for assessing impacts in the factual and counterfactual climate conditions. As such, most studies did not explicitly consider how vulnerability changes over time when assessing anthropogenic health impacts across long periods of time (Vicedo-Cabrera et al. 2021). These assessments argued that average risk across the study period would provide more robust and reliable estimates. We evaluated the effect of this assumption by comparing results generated using a single exposure-response calculated for the full analysis period with results that account for how mortality risk changed over 1969-2018.

Our overall results are consistent when generated using a single (full-period) and time-varying exposure-response associations. Across the full analysis period, heat-related mortality attributable to climate change was 1.4% of all-cause mortality when calculated with both time-varying and constant exposure-response associations (5–95% empirical confidence intervals are 0.1–2.9% for time-varying exposure-response association, and 0.2–2.7% for a constant exposure-response association), supporting the approach of previous analyses that used one exposure-response association for the full time series (Vicedo-Cabrera et al. 2021). However, we found that using a constant exposure-response association underestimates heat-related mortality at the start of the timeseries, when the relationship between temperature and mortality risk is steeper, and overestimates heat-related mortality in the period since 2004, as risk was reduced, as detailed below (Fig. 3a, Fig. S2). In our case study, our time-varying exposure-response association finds that the fraction of all-cause mortality that is attributable to heat is 4.20% (1.41–6.69%) in the observed climate of 1969–1985, compared with 3.88% (1.08–6.57%) for the constant exposure-response association. However, for 2004–2018, the heat-attributable fraction of mortality is Lower under the time-varying exposure-response association, at 4.83% (2.08–7.24%), compared with 6.32% (3.41–9.27%) for the constant exposure-response association. In any case, rising global temperatures continues to increase the deaths attributable to human-induced warming as a proportion of all-cause mortality: when using the single exposure-response association, from 0.68% (−0.79−1.51%) in 1969–1985, rising to 1.34% (0.05–2.77%) in 1986–2003 and 1.97% (1.2–3.94%) in 2004–2018.

Using the method described above, we then estimated the effect of changes in vulnerability on heat-related mortality by deriving exposure-response associations from all-cause summer mortality for each of three periods (1969–1985, 1986–2003, and 2004–2018) (Fig. S3). We assess the impacts of changes in vulnerability over time by comparing our results under a ‘constant vulnerability’ scenario in which the exposure-response association calculated for 1986–2003 is also applied to 2004–2018 with the findings in which vulnerability changes over time (‘evolving vulnerability’ scenario). Retaining the same exposure-response association across these sequential time periods assumes no changes in population structure or other factors that determine mortality risk. Similar approaches have been used outside of climate change attribution research, for instance by using age-stratified mortality associations with temperature (de Schrijver et al. 2022).

Under the ‘constant vulnerability’ scenario, in which these changes do not take place, an additional 730 deaths occurred over 2004–2018, relative to the ‘evolving vulnerability’ scenario when, in each case, mortality was calculated based on observed temperatures. The change in the exposure-response relationship from 2004 onwards thus caused an 29% reduction in heat-related mortality over the period 2004–2018 relative to the mortality expected in the absence of these changes (Table S6). In both the constant and evolving vulnerability scenarios, heat-related mortality attributable to climate change rose throughout the study period, with reductions in vulnerability limiting the extent of this increase (Table 1; Fig. 3c).

**Table 1:** Mean annual heat-related mortality attributable to anthropogenic climate change for the three periods considered in the analysis and for the three exposure-response scenarios. In the ‘evolving vulnerability’ scenario, a time-varying exposure response association was calculated based on observed temperatures and mortality for each of the three periods. In the ‘constant vulnerability’ scenario, the exposure-response association for 1986–2003 from the ‘evolving vulnerability’ scenario was also used for 2004–2018. In the ‘single exposure-response’ scenario, the exposure-response association was calculated based on observed mortality and temperature data from the full period (1969–2018). Central estimates and 5–95% confidence intervals were provided for the mortality values.

Scenario	Mean annual heat-related mortality attributable to anthropogenic climate change		
	1969–1985	1986–2003	2004–2018
Evolving vulnerability	19 (−24−47)	37 (1–79)	47 (21–92)
Constant vulnerability	19 (−24−47)	37 (1–79)	58 (29–109)
Single exposure-response	16 (−18−35)	33 (1–69)	48 (29–97)

### Attributable mortality within and outside heatwave periods

**Fig. 4:**
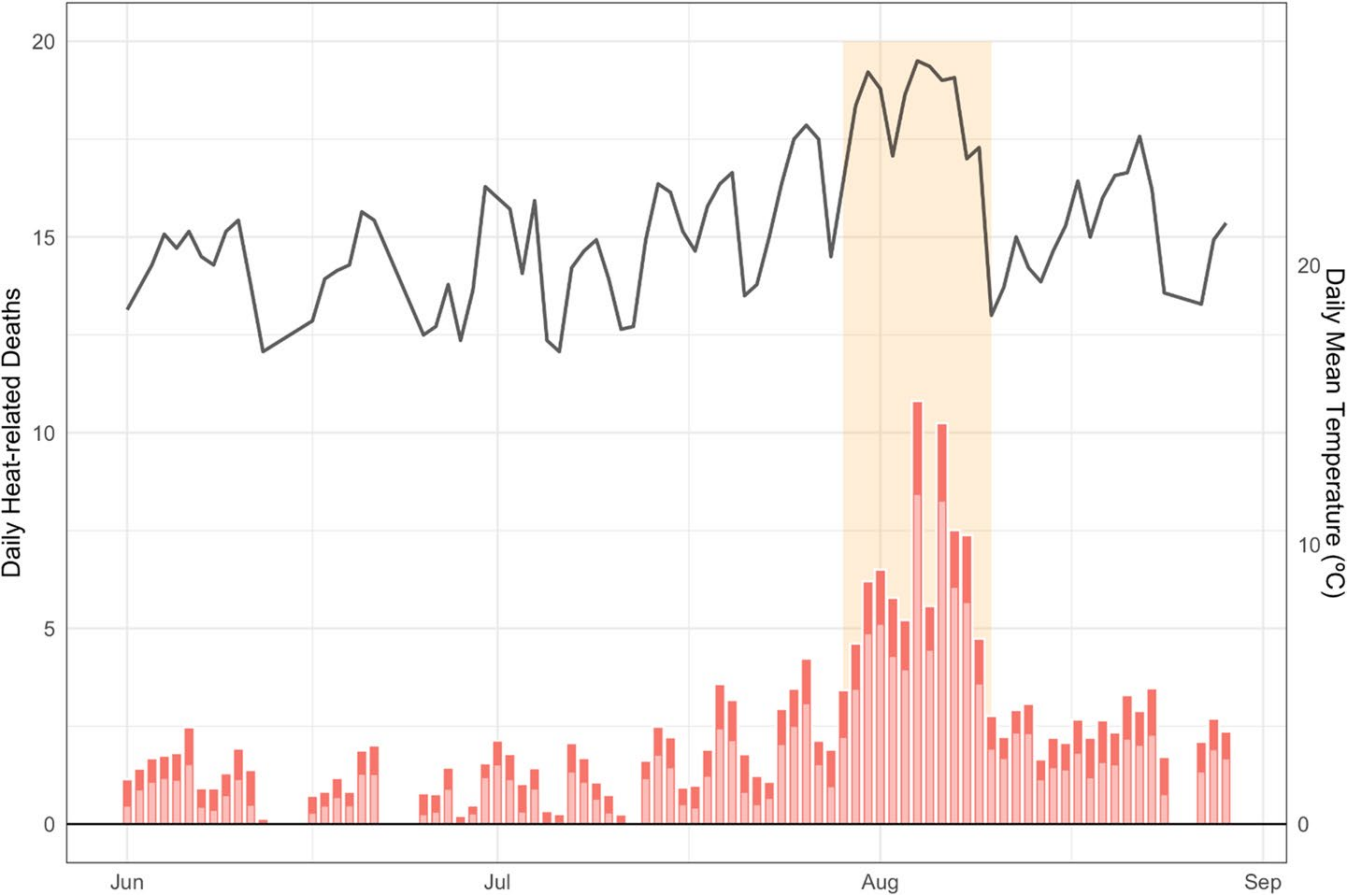
Observed daily-mean temperatures (black) and heat-related mortality (red bars) showing the portion attributable to anthropogenic climate change (dark red) for the Canton of Zürich, June – August 2018. Daily heat-related deaths are represented by the height of the bar, with the dark red segment corresponding to the number of daily deaths attributable to climate change and lighter red representing those deaths that would have been expected to occur in the counterfactual conditions where the effect of human-induced climate change is excluded.

We focus our heatwave case study on the summer of 2018, a period of then-record-breaking heat across much of Europe (Rousi et al. 2023). Human influence was found in previous studies to Have increased the intensity and likelihood of the summer 2018 heatwave in northern and southern Europe (Barriopedro et al. 2020; Yiou et al. 2020). For the summer (June – August) of 2018, we estimate that 86 (55–113, 5–95% empirical confidence intervals) of 208 (111–301, 5–95% empirical confidence intervals) heat-related deaths in the Canton of Zürich are attributed to anthropogenically-driven temperature rise (i.e., 41%), based on the temperature-mortality relationship derived for 2004–2018. We define the heatwave period as 29 July – 10 August, the period of highest temperatures. Across these 12 days (13% of summer), 83 (46–116) heat-related deaths occurred, with 22 (−3−46) attributable to climate change (27% of heat-related deaths, Fig. 4).

Our findings provide an empirical demonstration that climate change increases heat-related mortality on almost all summer days. While mortality peaks during heatwaves, much of the heat-related mortality attributable to climate change also occurs outside of heatwaves.

## Discussion

Our results show a substantial burden of heat-related mortality attributable to climate change in Zürich. As in previous studies, we used a single exposure-response relationship derived from all observed mortality and temperature series representing the average vulnerability across the study period (Vicedo-Cabrera et al. 2021). Using the full timeseries reduces uncertainty in the exposure-response association, especially in locations with a low number of observations. However, the temperature-mortality relationship evolves over time due to changes in demographic (de Schrijver et al. 2022), physiological, behavioural, socioeconomic, and infrastructural factors (Mitchell 2021). A long daily-mortality dataset allows us to subset the study period to assess changes in exposure-response associations over time. This allowed us to evaluate the previously used approach of using a single exposure-response relationship for the full time series and examine the effect of changing vulnerability over time. Nevertheless, we do not disaggregate the effects of specific drivers of vulnerability. Future work could attempt to assess the contributions of changing demographic factors, improved health systems, introduction of heat response plans, changes in behaviour, changes in urban green space and the built environment to burdens of heat-related mortality.

Previous research quantified heat-related mortality attributable to anthropogenic climate change during heatwave periods alone (Mitchell 2016; Guo et al. 2018), across full summers (Mitchell et al. 2016) and over multiple years (Vicedo-Cabrera et al. 2021). The methods applied here address a limitation of these previous studies. Some analyses quantify long-term impacts but cannot accurately assess specific short-term events within the study period. Studies that quantify attributable heat-related mortality using changing probabilities of observed meteorological conditions due to climate change have been applied to events of limited duration but not long-term effects. Our approach to estimating counterfactual temperatures provides fungible results for heatwaves and longer periods. This allows us to conduct a long-term analysis that can be subset to provide results that for individual events within that longer timeseries.

Our climatological and epidemiological case study analyses yield three main findings. First, in the Canton of Zürich, 1,683 (270-3,279) summer heat-related deaths were attributed to anthropogenic climate change in 1969–2018. The range of different mortality estimates across observations, models and reanalysis datasets underlines the importance of using multiple lines of evidence to represent uncertainty in climate-change-attributable mortality. Second, reductions in vulnerability counteracted some impacts of rising temperatures, limiting the increase in heat-related mortality. Nevertheless, substantial heat impacts were still observed. Zürich enjoys advantages in its capacity to adapt to heat effects compared with many locations worldwide due to high quality housing, health and social services, and economic prosperity. Empirical evidence for the effectiveness of adaptation is limited (Berrang-Ford et al. 2021). The results above demonstrate the effects of vulnerability changes, and therefore the potential effectiveness of adaptation measures. Implementing adaptation measures may incur costs in addition to the benefits, in terms of avoided mortality, detailed here, although these were beyond the scope of our analysis.

Third, heat-related mortality occurs at highest rates at the hottest temperatures. However, outside of heatwaves, the proportion of summer mortality attributable to anthropogenic climate change is higher (27% during the hottest 10% of summer days, versus 41% across summer 2018): climate change amplifies mortality throughout warm seasons. This finding is not, in fact, counterintuitive. Because the temperature-mortality relationship is steepest at high temperatures, absolute values of climate-change-attributable heat-related mortality are highest on the hottest days. However, on the more numerous days that are cooler but still above the minimum mortality temperature, climate change is responsible for a larger proportion of heat-related deaths. This is because the attributable warming accounts for a larger proportion of the difference between the observed and minimum-mortality temperature than on hot days. Analyses that focus on heatwaves alone will not capture the full effect of anthropogenic climate change on heat-related deaths.

Previous research found that globally 0.6% of warm-season all-cause mortality (37% of heat-related mortality) was attributable to anthropogenic climate change over 1991–2018 (0.74% and 31.3% respectively for Switzerland, based on seven cities and one metropolitan area) (Vicedo-Cabrera et al. 2021). Here, heat-related mortality attributable to climate change was calculated as the difference between growing numbers of heat-related deaths occurring at increasingly common high temperatures and reduced numbers of deaths occurring at decreasingly common moderate temperatures (the difference between the areas under the curves in Fig. 2c). Observed trends in heat-related mortality might be partially affected by changes in winter mortality because more potentially vulnerable people survive the winter months (Ha et al. 2011; Armstrong et al. 2017), but they are not accounted for in this analysis and it is unclear to what extent this may be the case in the context of Zürich.

Previous attribution studies did not attempt to disaggregate quantitatively the effects of physical hazards between climate change, and other factors such as changing societal exposure and vulnerability (Jézéquel et al. 2024). Societal changes that may be unrelated to climate, such as improvements in public health systems, affect vulnerability to physical hazards, as does climate change adaptation. Challenges in disaggregating these factors and the absence of empirical evidence of how risk would have changed in the absence of climate change complicates identifying which drivers lead to reduced vulnerability. Due to these challenges, most attribution studies estimate mortality risk in presence of climate change and then apply the same exposure-response association to a counterfactual scenario where climate change is excluded. However, vulnerability to extreme weather events may have evolved differently in the absence of climate change. Here, we apply a simple approach to account for the effects of changes in vulnerability.

The reduced mortality that we find to be associated with changes in vulnerability could represent effects of changes in demographics, access to and quality of public health and healthcare systems, physiological acclimatization, behaviour, access to blue and green spaces, and infrastructure, to name just a few. Our approach does not facilitate disaggregation of the effects of specific drivers of the change in population sensitivity to heat. The Canton of Zürich has implemented limited measures for reducing heat impacts beyond those introduced at the Federal level (Ragettli and Röösli 2019), including information campaigns advising vulnerable populations on safe behaviour during heatwaves (Ragettli et al. 2017). Concurrently, population ageing in Zürich increased vulnerability to heat (de Schrijver et al. 2022).

Our results show that vulnerability to heat in Zürich has changed substantially over time. This change in vulnerability is likely to include the effect of adaptation measures, such as changes in behaviour and the availability of air conditioning, as well as factors such as improved healthcare, all of which can reduce the burden of mortality associated with high temperatures. These results contrast with previous research that compared exposure-response associations for successive periods of time to evaluate England’s heatwave plan and found very little change in the temperature-risk relationship following its introduction (Williams et al. 2019). Nevertheless, substantial climate-change-attributable heat-related mortality continues to occur in Zürich. Under both ‘constant vulnerability’ and ‘evolving vulnerability’ scenarios, attributable heat-related mortality constitutes an increasing portion of all-cause summer mortality at higher levels of regional warming (Fig. 3c).

We found that an average of 19 (−24−47) heat-related deaths attributable to anthropogenic climate change occurred each summer in 1969–1985, rising to 47 (21–92) since 2004, a 250% increase over a period when population increased by just 40% (Federal Statistical Office 2024; Wikipedia 2025). A simple method for allocating contributions to impacts that is commonly used in legal settings is the ‘market-share approach’ that estimates individual entities’ contributions to losses as the product of the entity’s proportional contribution to greenhouse gas emissions and the total impacts attributable to climate change (Stuart-Smith et al. 2021). Applying this approach here indicates that cumulative greenhouse gas emissions of each of the top six highest-emitting investor and state-owned companies globally (Heede 2014) caused, on average, at least one additional death per summer in Zürich since 2004 (estimates for the ten highest-emitting companies globally, for 1969–2018 are provided in Table S7). Similar findings would be expected for many other locations worldwide.

Above, we explain that different approaches to conducting climate change attribution studies have different applications depending on the relationship between climatic hazards and their impacts. The method described here does not require additional data relative to the commonly used approach described in Vicedo-Cabrera et al. (2021). However, our analyses do require data that are not available worldwide, including daily all-cause mortality and temperature observations. Limited availability and quality of granular health data and long-term climate observations (although satellite observations can fill this gap in recent years for weather observations) present challenges for deriving location-specific relationships between climate variables and health outcomes, or in evaluating human influence on these climate variables.

Our results are consistent with previous analyses (Vicedo-Cabrera et al. 2021). They demonstrate the scale of impacts already occurring because of o5bserved climate change and indicate the risk of worsening impacts under further heating, with global implications. The approach described could be applied elsewhere and adapted to evaluate the effect of climate change on other health risks or economic impacts and could be compared to exposure over time to other known hazards such as tobacco smoke for further contextualization. The methods employed here could be adjusted to make greater use of reanalysis data in lieu of direct meteorological observations, in regions where these are limited or absent, and estimate exposure-response associations based on available health data in conjunction with socioeconomic, institutional, climatological, demographic, and environmental information. Further methodological developments are required to enable detection of hard limits to adaptation to enable for evidence-based adaptation planning. These methodological alterations could support evidence-based actions to reduce climate change impacts on health such as improved early warning systems to enable early action, strengthened health system preparedness, and improved health workforce and emergency response capacity. These impacts disproportionately affect vulnerable groups, such as those living in poverty and urban areas (The Lancet 2022). Nevertheless, as the health effects of climate change continue to worsen globally, location-specific analyses like the case study reported here provide important data points for understanding the extent of climate change impacts on human health and the consequences of vulnerability and its changes over time. Results like ours underline the heightened importance of work to prepare, adapt, respond, and improve resilience in the face of rising exposure to worsening impacts of climate change.

## Supplementary Information

Below is the link to the electronic supplementary material.

ESM1 (454 KB)

## Data Availability

Temperature observations for Zürich/Fluntern can be downloaded from the KNMI Climate Explorer at http://climexp.knmi.nl/ (last accessed: 02.01.2024). ERA5 reanalysis data are openly available from the Copernicus Climate Change Service at https://cds.climate.copernicus.eu/#!/home (last accessed: 08.05.2025), MERRA2 from NASA Goddard Earth Sciences (GES) Data and Information Services Center (DISC) at https://disc.gsfc.nasa.gov/datasets/M2TMNXSLV_5.12.4/summary (last accessed: 02.01.2024), and Berkeley Earth from http://berkeleyearth.org/data/ (last accessed: 08.05.2025). All the CMIP6 model simulations are obtained from the CEDA Archive-part of NERC’s Environmental Data Service at https://catalogue.ceda.ac.uk/?q=CMIP6%26;sort_by=relevance%26;results_per_page=20 (last accessed: 08.05.2025). Daily mortality data are sourced from the Federal Office of Statistics (Switzerland) and include non-external causes only other than accidents (ICD-10 codes A00-R99, V01-V99, W00-X59) for the period 1969–2018. Mortality data is available at the official data repository of the University of Bern (BORIS) at 10.48620/90843.

## Code availability

Code for the analysis of climate data will be available upon request to the corresponding author. Code for the epidemiological analysis is available at https://github.com/anavica/attrzurich_stuartsmith_etal.git.
