# Supplementary material for: Refining methods for attributing health impacts to climate change: a heat-mortality case study in Zürich
Source: Clim Change. 2025 Sep 10;178(9):165. doi: 10.1007/s10584-025-04011-5 (PMC12423209; doi:10.1007/s10584-025-04011-5)
Supplement: Supplementary file 1 — ESM1 (454 KB) [file 10584_2025_4011_MOESM1_ESM.docx]

**Refining methods for attributing health impacts to climate change: a heat-mortality case study in Zürich**

**Supplementary information**

Supplementary Figures and Tables


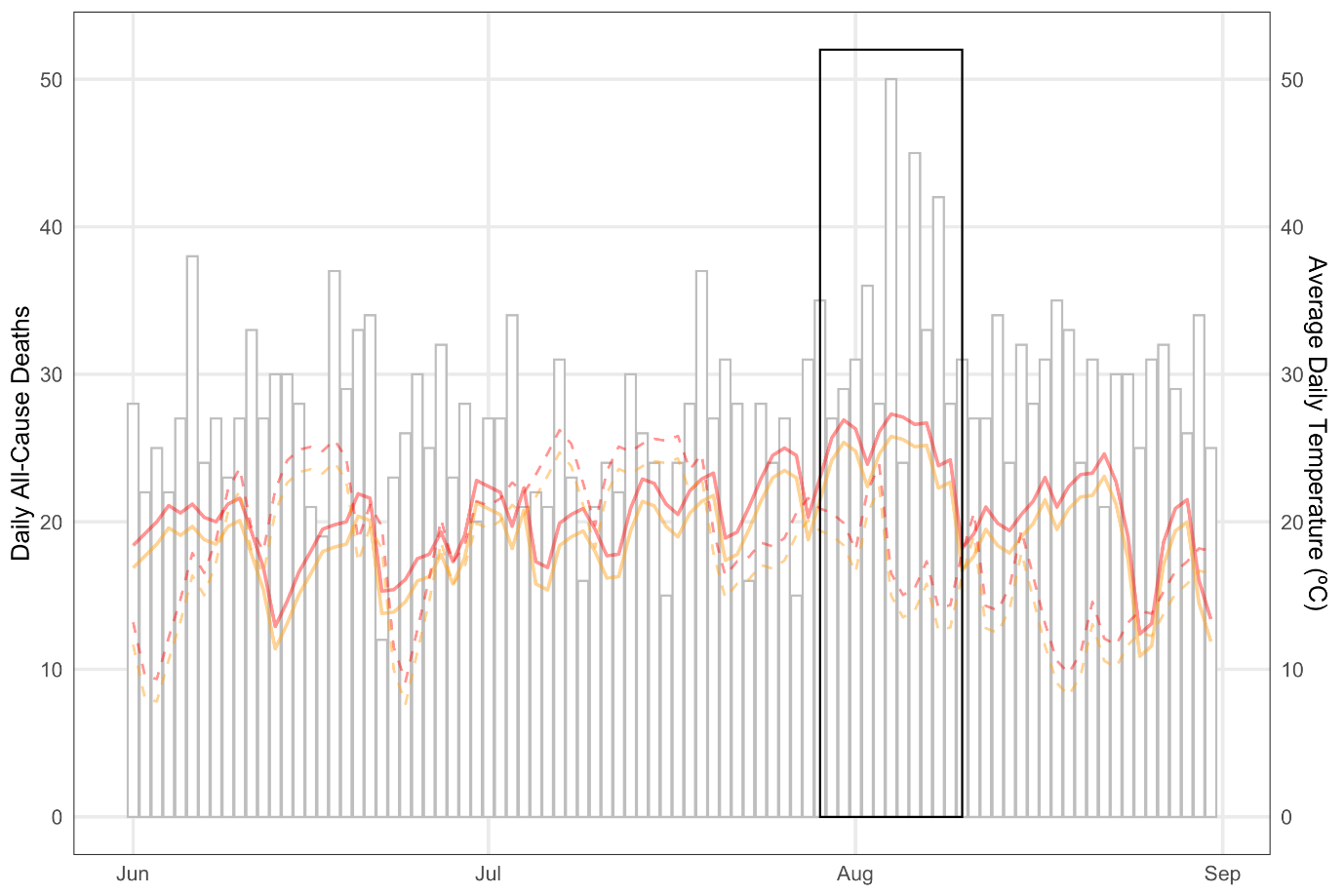


Fig. S1.

**Daily all-cause mortality and daily-mean temperatures for the historical (red) and counterfactual (orange) scenarios based on the method applied in this study (solid lines) and the approach taken by Vicedo-Cabrera et al. (2021; dashed lines) and for the CMCC-ESM2 model in each case.** The dashed lines show historical and historical-natural data from the CMCC-ESM2 model simulations, bias corrected using the same statistical approach (Hempel et al. 2013) applied in Vicedo-Cabrera et al. (2021).


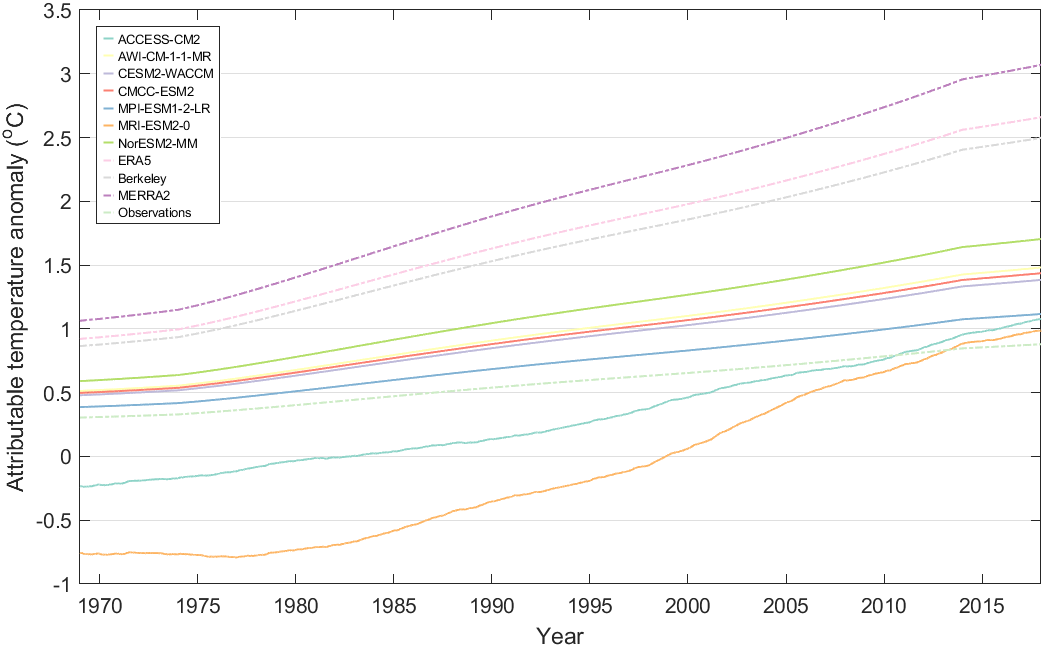


Fig. S2.

**Temperature anomaly attributable to anthropogenic climate change for 1969-2018 in climate-model timeseries, reanalysis and station observations.** Temperature anomalies are the difference between factual and counterfactual timeseries in each of the observed and model datasets, based on a baseline period of 1989-2018. Observation-based (reanalysis and station records) data are shown as dashed lines, whereas model data are presented as solid lines. The negative anomaly seen in two of the model timeseries during the early portion of the displayed data (ACCESS-CM2 and MRI-ESM2-MM) is explained in Methods (‘Constructing the counterfactual temperatures’). By the end of the study period, all models’ attributable temperature anomalies lie within the range of the observation-based datasets.


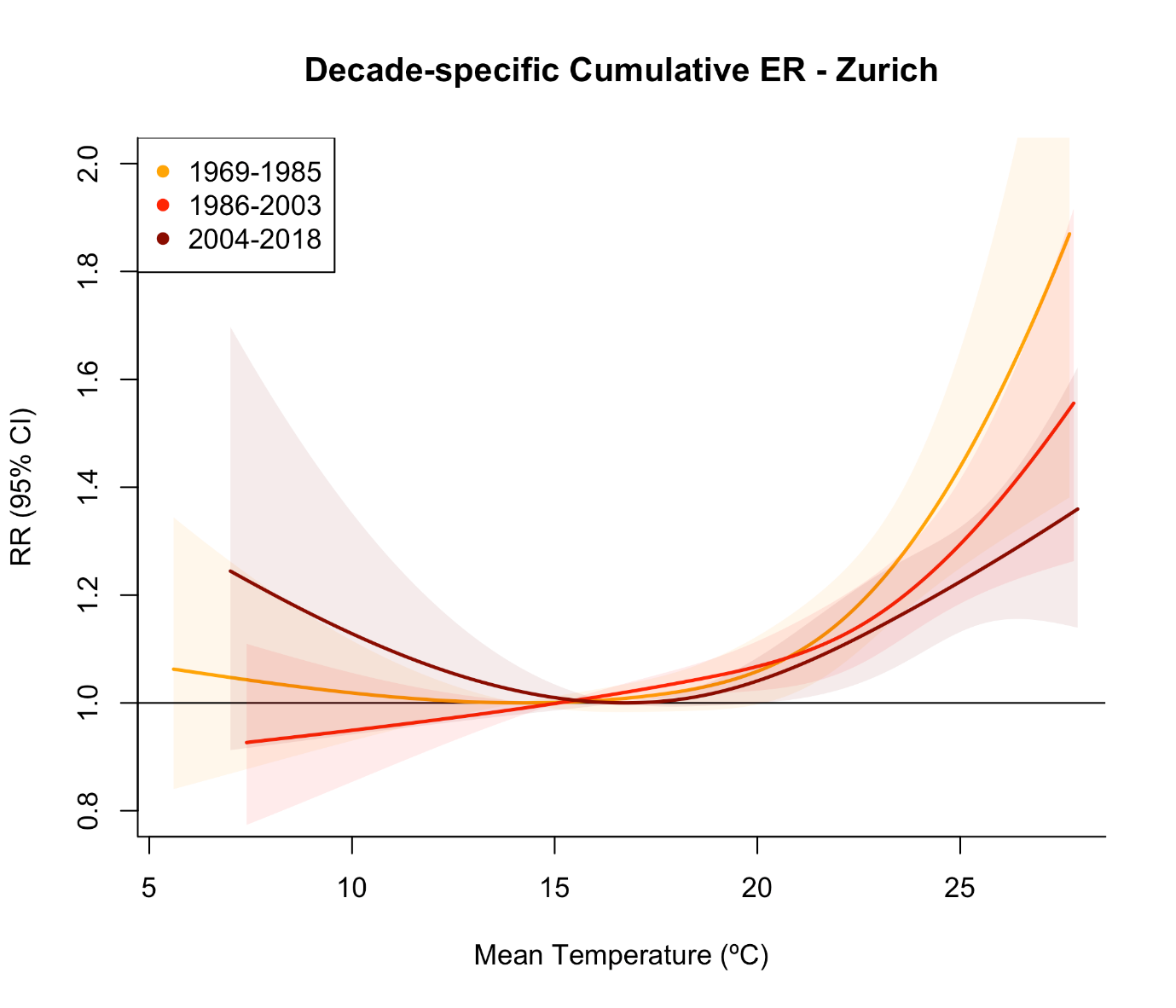


Fig. S3.

**Exposure-response associations derived from observed daily-mean temperature and mortality, for 1969-1985, 1986-2003, and 2004-2018.** 5-95% empirical confidence intervals are represented by the shaded areas.

Table S1.

**CMIP6 models used in analysis showing those selected in the evaluation step and those excluded following evaluation.**

| Models selected in analysis | Non-selected models |
| --- | --- |
| ACCESS.CM2 | ACCESS-ESM1-5 |
| AWI-CM-1-1-MR | CESM2 |
| CESM2-WACCM | CEMCC-CM2-SR5 |
| CMCC-ESM2 | FGOALS-g3 |
| MPI-ESM1-2-LR | GFDL-ESM4 |
| MRI-ESM2.0 | INM-CM4-8 |
| NorESM2-MM | INM-CM5-0 |
|  | MIROC6 |
|  | MIROC-ES2L |
|  | NorESM2-LM |
|  | TaiESM1 |

Table S2.

**Detailed information for the climate models used for analysis in study.**

| Climate model | Institute | Spatial resolution (# of grid cells longitude by latitude) | Spatial resolution (degrees longitude by latitude) | Experimental ID used |
| --- | --- | --- | --- | --- |
| ACCESS-CM2 | CSIRO (Commonwealth Scientific and Industrial Research Organisation, Australia), ARCCSS (Australian Research Council Centre of Excellence for Climate System Science) | 192 x 144 | 1.875 x 1.25 | historical  SSP5-8.5  hist-nat |
| AWI-CM-1-1-MR | Alfred Wegener Institute, Helmholtz Centre for Polar and Marine Research, Germany | 384 x 192 | 0.94x0.94 | historical  SSP5-8.5 |
| CESM2-WACCM | National Center for Atmospheric Research, Climate and Global  Dynamics Laboratory, USA | 288 x 192 | 1.25 x 0.94 | historical  SSP5-8.5 |
| CMCC-ESM2 | Fondazione Centro Euro-Mediterraneo sui Cambiamenti Climatici,  Italy | 288 x 192 | 1.25 x 0.94 | historical  SSP5-8.5 |
| MPI-ESM1-2-LR | Max Planck Institute for Meteorology, Alfred WegenerInstitute, Deutsches Klimarechenzentrum and DeutscherWetterdienst, Germany | 192 x 96 | 1.875x1.875 | historical  SSP5-8.5 |
| MRI-ESM2-0 | Meteorological Research Institute, Tsukuba, Ibaraki 305-0052, Japan | 320 x 160 | 1.125x1.125 | historical  SSP5-8.5  hist-nat |
| NorESM2-MM | NorESM Climate modeling Consortium consists of CICERO, MET-Norway, NERSC, NILU, UiB, UiO and UNI, Norway. | 288 x 192 | 1.25 x 0.94 | historical  SSP5-8.5 |

Table S3.

**Heat-related mortality attributable to anthropogenic climate change for each model and observation-based dataset, 1969-2018.** ‘Observations synthesis’ [OS], ‘Model synthesis’ [MS] and ‘Full synthesis’ [FS] values are calculated as geometric means of the observations and reanalysis (ERA5, Berkeley Earth & MERRA2) [OS], models [MS], and observations, reanalysis and models [FS], respectively.

| Dataset (model run or reanalysis) | Central estimate | 5% confidence level | 95% confidence level |
| --- | --- | --- | --- |
| ACCESS.CM2 | 936 | 745 | 1121 |
| AWI.CM.1.1.MR | 1656 | 1317 | 1982 |
| CESM2.WACCM | 1560 | 1241 | 1867 |
| CMCC.ESM2 | 1612 | 1283 | 1930 |
| MPI.ESM1.2.LR | 1285 | 1024 | 1534 |
| MRI.ESM2.0 | 314 | 171 | 464 |
| NorESM2.MM | 1871 | 1478 | 2245 |
| Station observations | 1032 | 822 | 1228 |
| ERA5 | 2701 | 2057 | 3284 |
| Berkeley Earth | 2570 | 1969 | 3120 |
| MERRA2 | 3014 | 2280 | 3691 |
| Observations synthesis | 2323 | 899 | 3493 |
| Model synthesis | 1317 | 244 | 2072 |
| Full synthesis | 1683 | 270 | 3279 |

Table S4.

**Percentage of all-cause summer (June-August) mortality attributable to the impact of anthropogenic climate change on temperatures, 1969-2018.** ‘Observations synthesis’ [OS], ‘Model synthesis’ [MS] and ‘Full synthesis’ [FS] values are calculated as geometric means of the observations and reanalysis (ERA5, Berkeley Earth & MERRA2) [OS], models [MS], and observations, reanalysis and models [FS], respectively.

| Dataset | Central estimate (%) | 5% confidence level (%) | 95% confidence level (%) |
| --- | --- | --- | --- |
| ACCESS.CM2 | 0.78 | 0.62 | 0.93 |
| AWI.CM.1.1.MR | 1.37 | 1.09 | 1.64 |
| CESM2.WACCM | 1.29 | 1.03 | 1.55 |
| CMCC.ESM2 | 1.34 | 1.06 | 1.60 |
| MPI.ESM1.2.LR | 1.06 | 0.85 | 1.27 |
| MRI.ESM2.0 | 0.26 | 0.14 | 0.38 |
| NorESM2.MM | 1.55 | 1.22 | 1.86 |
| Station observations | 0.85 | 0.68 | 1.02 |
| ERA5 | 2.24 | 1.70 | 2.72 |
| Berkeley Earth | 2.13 | 1.63 | 2.58 |
| MERRA2 | 2.50 | 1.89 | 3.06 |
| Observations synthesis | 1.92 | 0.74 | 2.89 |
| Model synthesis | 1.09 | 0.20 | 1.72 |
| Full synthesis | 1.39 | 0.22 | 2.72 |

Table S5.

**Heat-related mortality calculated using the single exposure-response association (derived from all observed temperatures and mortality) and the time-varying exposure-response associations for the ‘evolving vulnerability’ and ‘constant vulnerability’ scenarios.** As described in the main text, the exposure-response association calculated for 1986-2003 is also applied to 2004-2018 in the ‘constant vulnerability’ scenario. Bracketed values are the 5-95% empirical confidence intervals.

|  | Single exposure-response | Evolving vulnerability | Constant vulnerability |
| --- | --- | --- | --- |
| Heat-related mortality (historical) | 6091 (2716-9476) | 5871 (2907-8551) | 6601 (3580-9252) |
| Heat-related mortality (counterfactual) | 4360 (988-7931) | 4134 (1558-7114) | 4705 (2015-7817) |
| Attributable heat-related mortality | 1683 (270-3279) | 1692 (160-3556) | 1853 (197-3828) |
| Heat-related mortality (% of all-cause mortality) | 5 (2.2-7.8) | 4.9 (2.4-7.1) | 5.5 (3-7.7) |
| Counterfactual heat-related mortality (% of all-cause mortality) | 3.6 (0.8-6.6) | 3.4 (1.3-5.9) | 3.9 (1.7-6.5) |
| Attributable heat-related mortality (% of all-cause mortality) | 1.4 (0.2-2.7) | 1.4 (0.1-2.9) | 1.5 (0.2-3.2) |
| Attributable portion of heat-related mortality (%) | 27.6 | 28.8 | 28.1 |

Table S6.

**Annual mean heat-related mortality in the three periods over our analysis under the three exposure-response scenarios.** The time-varying exposure-response was calculated based on observed temperature and mortality data for each of the three periods, the single exposure-response association is calculated for the full period 1969-2018, and the ‘constant vulnerability’ scenario is the time-varying exposure response but with the relationship calculated for 1986-2003 also applied to 2004-2018. Bracketed values are the 5-95% empirical confidence intervals

|  | 1969-1985 | 1986-2003 | 2004-2018 |
| --- | --- | --- | --- |
| Historical, ‘evolving vulnerability’ | 94 (30-149) | 141 (84-195) | 116 (49-174) |
| Counterfactual, ‘evolving vulnerability’ | 74 (14-144) | 104 (50-167) | 68 (16-124) |
| Attributable heat-related mortality (‘evolving vulnerability’) | 19 (-24-47) | 37 (1-79) | 47 (21-92) |
| Historical, single exposure response | 87 (23-148) | 130 (61-200) | 152 (81-224) |
| Counterfactual, single exposure response | 70 (11-138) | 93 (23-170) | 96 (24-171) |
| Attributable heat-related mortality (singe exposure response) | 16 (-18-35) | 33 (1-69) | 48 (29-97) |
| Historical, ‘constant vulnerability’ | 94 (30-149) | 141 (84-195) | 164 (104-220) |
| Counterfactual, ‘constant vulnerability’ | 74 (14-144) | 104 (50-167) | 106 (49-165) |
| Attributable heat-related mortality (‘constant vulnerability’) | 19 (-24-47) | 37 (1-79) | 58 (29-109) |

Table S7.

**Cumulative 1854-2010 scope 1,2, & 3 emissions attributable to the ten highest-emitting investor- and state-owned companies, the proportion of historical anthropogenic greenhouse gas emissions attributable to these actors, and the estimated heat-related mortality attributable to each company for the Canton of Zürich over 1969-2018.** Historical emissions data are sourced from Heede (2014).

| Company | Cumulative emissions 1854-2010 (GtCO_2_e) | Percent of global anthropogenic emissions (1751-2010) | Attributable heat-related mortality, 1969-2018 (5-95% confidence intervals) |
| --- | --- | --- | --- |
| Chevron | 51.1 | 3.52 | 59 (10-115) |
| ExxonMobil | 46.7 | 3.22 | 54 (9-106) |
| Saudi Aramco | 46.0 | 3.17 | 53 (9-104) |
| BP | 35.8 | 2.47 | 42 (7-81) |
| Gazprom | 32.1 | 2.22 | 37 (6-73) |
| Royal Dutch / Shell | 30.8 | 2.12 | 36 (6-70) |
| National Iranian Oil Company | 29.1 | 2.01 | 34 (5-66) |
| Pemex | 20.0 | 1.38 | 23 (4-45) |
| Conoco Philips | 16.9 | 1.16 | 20 (3-38) |
| Petroleos de Venezuela | 16.2 | 1.11 | 19 (3-36) |

**Table S8**

**Equilibrium Climate Sensitivity of climate models used in analysis as compared with the full set of CMIP6 Earth system models.**

| Model | Equilibrium Climate Sensitivity (°C) | Source |
| --- | --- | --- |
| ACCESS-CM2 | 4.7 | Meehl, G. A., Senior, C. A., Eyring, V., Flato, G., Lamarque, J. F., Stouffer, R. J., et al. (2020). Context for interpreting equilibrium climate sensitivity and transient climate response from the CMIP6 Earth System Models. *Science Advances*, **6**(26), eaba1981. <https://doi.org/10.1126/sciadv.aba1981> (Meehl et al. 2020) |
| AWI-CM-1-1-MR | 3.2 | Meehl, G. A., Senior, C. A., Eyring, V., Flato, G., Lamarque, J. F., Stouffer, R. J., et al. (2020). Context for interpreting equilibrium climate sensitivity and transient climate response from the CMIP6 Earth System Models. *Science Advances*, **6**(26), eaba1981. <https://doi.org/10.1126/sciadv.aba1981> (Meehl et al. 2020) |
| CESM2-WACCM | 4.8 | Meehl, G. A., Senior, C. A., Eyring, V., Flato, G., Lamarque, J. F., Stouffer, R. J., et al. (2020). Context for interpreting equilibrium climate sensitivity and transient climate response from the CMIP6 Earth System Models. *Science Advances*, **6**(26), eaba1981. <https://doi.org/10.1126/sciadv.aba1981> (Meehl et al. 2020) |
| CMCC-ESM2 | 3.57 | Lovato, T., Peano, D., Butenschön, M., Materia, S., Iovino, D., Scoccimarro, E., et al. (2022). CMIP6 simulations with the CMCC earth system model (CMCC-ESM2). Journal of Advances in Modeling Earth Systems, 14(3), e2021MS002814. <https://doi.org/10.1029/2021MS002814> (Lovato et al. 2022) |
| MPI-ESM1-2-LR | 3.0 | Meehl, G. A., Senior, C. A., Eyring, V., Flato, G., Lamarque, J. F., Stouffer, R. J., et al. (2020). Context for interpreting equilibrium climate sensitivity and transient climate response from the CMIP6 Earth System Models. *Science Advances*, **6**(26), eaba1981. <https://doi.org/10.1126/sciadv.aba1981> (Meehl et al. 2020) |
| MRI-ESM2-0 | 3.2 | Meehl, G. A., Senior, C. A., Eyring, V., Flato, G., Lamarque, J. F., Stouffer, R. J., et al. (2020). Context for interpreting equilibrium climate sensitivity and transient climate response from the CMIP6 Earth System Models. *Science Advances*, **6**(26), eaba1981. <https://doi.org/10.1126/sciadv.aba1981> (Meehl et al. 2020) |
| NorESM2-MM | 2.5 | Seland, Ø., Bentsen, M., Olivié, D., Toniazzo, T., Gjermundsen, A., Graff, L. S., Debernard, J. B., Gupta, A. K., He, Y.-C., Kirkevåg, A., Schwinger, J., Tjiputra, J., Aas, K. S., Bethke, I., Fan, Y., Griesfeller, J., Grini, A., Guo, C., Ilicak, M., … Schulz, M. (2020). Overview of the Norwegian Earth System Model (NorESM2) and key climate response of CMIP6 DECK, historical, and scenario simulations. *Geoscientific Model Development*, *13*(12), 6165–6200. <https://doi.org/10.5194/gmd-13-6165-2020>. (Seland et al. 2020) |
| Selected model mean | 3.567 |  |
| CMIP6 multimodel mean (39 models) | 3.7 | Meehl, G. A., Senior, C. A., Eyring, V., Flato, G., Lamarque, J. F., Stouffer, R. J., et al. (2020). Context for interpreting equilibrium climate sensitivity and transient climate response from the CMIP6 Earth System Models. *Science Advances*, **6**(26), eaba1981. <https://doi.org/10.1126/sciadv.aba1981> (Meehl et al. 2020) |
| CMIP6 model range | 1.8-5.6 | Meehl, G. A., Senior, C. A., Eyring, V., Flato, G., Lamarque, J. F., Stouffer, R. J., et al. (2020). Context for interpreting equilibrium climate sensitivity and transient climate response from the CMIP6 Earth System Models. *Science Advances*, **6**(26), eaba1981. <https://doi.org/10.1126/sciadv.aba1981> (Meehl et al. 2020) |

**References**

Heede R (2014) Tracing anthropogenic carbon dioxide and methane emissions to fossil fuel and cement producers, 1854–2010. Clim Change 122:229–241. https://doi.org/10.1007/s10584-013-0986-y

Hempel S, Frieler K, Warszawski L, et al (2013) A trend-preserving bias correction – the ISI-MIP approach. Earth System Dynamics 4:219–236. https://doi.org/10.5194/esd-4-219-2013

Lovato T, Peano D, Butenschön M, et al (2022) CMIP6 Simulations With the CMCC Earth System Model (CMCC‐ESM2). J Adv Model Earth Syst 14:e2021MS002814. https://doi.org/10.1029/2021MS002814

Meehl GA, Senior CA, Eyring V, et al (2020) Context for interpreting equilibrium climate sensitivity and transient climate response from the CMIP6 Earth system models. Sci Adv 6:. https://doi.org/10.1126/sciadv.aba1981

Seland Ø, Bentsen M, Olivié D, et al (2020) Overview of the Norwegian Earth System Model (NorESM2) and key climate response of CMIP6 DECK, historical, and scenario simulations. Geosci Model Dev 13:6165–6200. https://doi.org/10.5194/gmd-13-6165-2020

Vicedo-Cabrera AM, Scovronick N, Sera F, et al (2021) The burden of heat-related mortality attributable to recent human-induced climate change. Nat Clim Chang 11:492–500. https://doi.org/10.1038/s41558-021-01058-x
